# Eravacycline activity against clinical *S. aureus* isolates from China: in vitro activity, MLST profiles and heteroresistance

**DOI:** 10.1186/s12866-018-1349-7

**Published:** 2018-12-13

**Authors:** Fan Zhang, Bing Bai, Guang-jian Xu, Zhi-wei Lin, Gui-qiu Li, Zhong Chen, Hang Cheng, Xiang Sun, Hong-yan Wang, Yan-wei Chen, Jin-xin Zheng, Qi-wen Deng, Zhi-jian Yu

**Affiliations:** 1 Department of Infectious Diseases and Quality Control Center of Hospital Infection Management of Shenzhen, Shenzhen Nanshan People’s Hospital, Guang Dong Medical University, No 89, Taoyuan Road, Nanshan District, Shenzhen, 518052 China; 2 Shenzhen key laboratory for endogenous infections, Shenzhen Nanshan People’s Hospital, Shenzhen University school of medicine, No 89, Taoyuan Road, Nanshan District, Shenzhen, 518052 China; 3 Department of Tuberculosis, Shenzhen Nanshan Center for Chronic Disease Control, No 7, Huaming Road, Nanshan District, Shenzhen, 518054 China; 4 Key Laboratory of Medical Molecular Virology of Ministries of Education and Health, School of Basic Medical Sciences and Shanghai Public Health Clinical Center, Fudan University, Shanghai, 200032 China

## Abstract

**Background:**

Mortality rates for patients with *Staphylococcus aureus* (*S. aureus*) infections have improved only modestly in recent decades and *S. aureus* infections remain a major clinical challenge This study investigated the in vitro antimicrobial activity of erevacycline (erava) against clinical *S. aureus* isolates from China, as well as the heteroresistance frequency of erava and sequence types (STs) represented in the sample.

**Results:**

A sample of 328 non-duplicate clinical *S. aureus* isolates*,* including 138 methecillin-resistant (MRSA) and 190 methecillin-sensitive (MSSA) isolates, were collected retrospectively in China. Erava exhibited excellent in vitro activity (MIC_50_ ≤ 0.25 mg/L) against MRSA and MSSA, including isolates harboring Tet specific resistance genes. The frequency of erava heteroresistance in MSSA with erava MICs = 0.5 mg/L was 13.79% (4/29); no MRSA with erava MICs ≤0.5 mg/L exhibited heteroresistance. Heteroresistance- derived clones had no 30S ribosome subunit mutations, but their erava MICs (range, 1–4 mg/L) were suppressed dramatically in the presence of efflux protein inhibitors.

**Conclusions:**

Conclusively, erava exhibited excellent in vitro activity against *S. aureus*, however hints of erava heteroresistance risk and MIC creep were detected, particularly among MSSA with MICs of 0.5 mg/L.

**Electronic supplementary material:**

The online version of this article (10.1186/s12866-018-1349-7) contains supplementary material, which is available to authorized users.

## Background

Mortality rates for patients with *Staphylococcus aureus* (*S. aureus*) infections have improved only modestly in recent decades and *S. aureus* infections remain a major clinical challenge [1]. Indeed, multi-drug resistant *S. aureus* has been reported worldwide and its rates are increasing, particularly MRSA, where only last resort antibiotics can be used, such as tigecycline, daptomycin, and linezolid [2]. The recently developed antibiotic eravacycline (erava; a.k.a. TP-434), a synthetic fluorocycline, was shown to be active against numerous Gram-positive, Gram- negative, and anaerobic bacteria, including those with acquired tetracycline (Tet)-specific efflux pumps, ribosomal protection factors, and multi-drug resistance mechanisms [3–5]. However, data regarding the efficacy of erava against clinical *S. aureus* isolates, especially from China, are quite limited [3–7]. Moreover, studies showing excellent in vitro antimicrobial activity of erava against *S. aureus*, enterococci, and streptococci, among others, revealed the rare occurrence of antimicrobial resistance [3, 8].

The development of heteroresistance in bacteria under antibiotic pressure often leads to antimicrobial treatment failure, and vancomycin heteroresistance in *S. aureus* in particular has become a serious threat for the induction of resistance that can make treatment difficult [9]. Furthermore, in recent years, strains of several microbial species have exhibited troubling heteroresistance to tigecycline [10, 11], which is structurally similar to erava.

Emergent mutations in genes encoding several 30S ribosome components, including 16SrRNA and ribosomal S10, have been found to correlate with reduced susceptibility to tigecycline [8, 12–20]. Tigecycline resistance in *S. aureus* has been reported to correlate closely with overexpression of a MATE (multidrug and toxic compound extrusion) family efflux pump encoded by *mepA* in passaged mutants, and some *Escherichia coli* or *Enterococcus faecium* isolates expressing high levels of the Tet-specific resistance factors Tet(M), Tet(K), and Tet(B) have been found to have tigecycline minimum inhibitory concentration (MIC) values two to four-fold to that in the controls [8, 12]. Tigecycline nonsusceptibility and heteroresistance risk in several Gram-negative bacteria species have been reported to correlate with expression levels of several efflux pumps and cell envelope proteins [21–23]. To date, erava efficacy appears to be mostly unaffected by common Tet resistance factors [3, 4]. However, the potential effects of Tet resistance factors on the development of erava resistance or heteroresistance in *S. aureus,* including cross-resistance to tigeycline, needs to be further studied.

Carbonyl cyanide m-chlorophenylhydrazine (CCCP) is a protonophore that exhibits reversible binding and transmembrane transport of protons across the cell membrane, leading to membrane depolarization that can diminish the electrochemical gradient and thereby reduce ATP production by ATP synthase [10, 21–23]. It has been used to investigate how protonophore activity can affect the potency of critical reserve antibiotics, including carbapenem, colistin, and tigecycline [21–23]. Phe-Arg-β- naphthylamide (PAβN) is a highly active broad-spectrum inhibitor of bacterial resistance-nodulation-division efflux pumps [21]. It remains to be determined how CCCP and PAβN may affect erava MICs.

The overall aim of this study was to examine the characteristics of erava antimicrobial activity and potential risks of erava resistance mechanisms. Toward this aim, we assessed the in vitro activity of erava against clinical *S. aureus* isolates from China and estimated the frequency of erava heteroresistance with population analysis profiling (PAP). Multilocus sequence typing (MLST) was performed to identify the molecular genotype of the isolates in our sample. We then used polymerase chain reaction (PCR) amplification to detect mutations in genes encoding Tet target sites in heteroresistance-derived *S. aureus* clones. Finally, we employed CCCP and PAβN experimental assays to examine the role of cell envelope and efflux pump proteins in erava heteroresistance.

## Methods

### Bacteria isolates

A total of 328 non-duplicate clinical *S. aureus* isolates, including both methicillin-resistant (MRSA) and methicillin- susceptible (MSSA) *S. aureus* isolates, were collected retrospectively from Shenzhen Nanshan People’s Hospital, a tertiary hospital with 1200 beds in China, from 2010 through 2016. There were 138 MRSA isolates (105 from tracheal secretions, 22 from pus, and 11 from blood). There were 190 MSSA isolates (131 from tracheal secretions, 38 from pus, and 21 from blood).

### Antimicrobial susceptibility testing

Antimicrobial susceptibilities of *S. aureus* isolates to a standard panel of clinically important drugs, including amoxicillin/clavulanate, amikacin, erythromycin, ciprofloxacin, rifampicin, Tet, tobramycin, nitrofurantoin, quinupristin, were determined with the VITEK 2 system (bioMérieux, Marcy l’Etoile, France). Erava was obtained from The Medicines Company (AdooQ Bioscience, USA). MICs of erava, vancomycin, and linezolid were determined by the agar dilution method according to CLSI guidelines [24]. *S. aureus* ATCC29213 was used as a quality control organism. Because the CLSI guidelines do not provide recommended the MIC susceptibility breakpoints for erava against *S. aureus*, we adopted an MIC susceptibility breakpoint of 0.5 mg/L, the value recommended for tigecycline nonsusceptibility in Gram-positive bacteria and defined heteroresistance as growth in 1 mg/L erava [25, 26]. Previous comparisons indicated that erava was two- to four-fold more active than Tigecycline against common Gram-positive aerobic bacteria [3, 6]. We employed four erava MIC levels in our antimicrobial susceptibility analysis (in mg/L): ≤0.125, 0.25, 0.5, and 1.0.

### Population analysis profiling (PAP)

PAP experiments were performed as described previously [10, 23]. Briefly, 50-μL aliquots (~ 10^8^ colony forming units/ml) were spread onto Müller-Hinton agar plates containing serially diluted erava (in mg/L: 0.5, 1.0, 2.0, 3.0). Colonies were counted after 24 h of incubation at 37 °C. Erava heteroresistance was defined as the observation of subpopulations isolated from the erava-containing plates able to grow in the presence of 1.0 mg/L erava (detection limit, ≥ 5 colony forming units/plate with an erava MIC ≥1.0 mg/L). Two heteroresistance-derived colonies were selected from the plates randomly and their erava and tigecycline MICs were determined by agar dilution as described above for further analysis of resistance mechanism [10, 23]. Erava heteroresistance risk was assessed separately for isolates with erava MIC values of ≤0.125 mg/L, 0.25 mg/L, and 0.5 mg/L.

### PCR amplification for detection of Tet specific resistance genes and 30S ribosome subunit mutations

Genomic DNA was extracted from all clinical isolates and used as templates for PCR amplification in lysis buffer for microorganisms to direct PCR (Takara Bio Inc., Japan). PCR analysis was performed to detect tet (K), tet (L), tet (M), and tet (O) as described previously [27]. PCR analysis and sequence alignment were used to screen for the presence of mutations in the genes encoding 30S ribosome subunit components (i.e., five separate copies of the 16SrRNA gene, and the genes encoding the 30S ribosomal protein S3 and 30S ribosomal protein S10) [15].all Primers in this study was shown in Additional file 1: Table S1.

### Multilocus sequence typing (MLST)

MLST was performed by amplifying and sequencing seven housekeeping genes (*arcC*, *aroE*, *glpF*, *gmK*, *pta*, *tpi*, and *yqiL*) and Allele numbers and sequence types (STs) were assigned in accordance with the MLST database (http://saureus.mlst.net/) [28]. PCR products were sequenced by BGI (Shenzhen, China).

### Efflux inhibition assays

Efflux pump and cell envelope protein involvement in erava heteroresistance was evaluated by determining erava MICs by the agar dilution method in the presence of efflux pump inhibitors, namely CCCP (16 mg/L) and PaβN (50 mg/L)(both from Sigma). MIC reduction to ≤¼ of control levels was considered a significant inhibitory effect [21, 22].

## Results

### In vitro activity of erava against clinical *S. aureus* isolates

Both MRSA and MSSA isolates were highly susceptible to rifampicin, nitrofurantoin, and quinupristin, as evidenced by low resistance rates (Tables 1 and 2). All *S. aureus* isolates were susceptible to vancomycin and linezolid, and all MSSA were susceptible toamoxicillin/clavulanate and nitrofurantoin. As reported in detail in Table 1, most of the MRSA and MSSA isolates had erava MICs at the ≤0.125 mg/L and 0.25 mg/L levels. The frequency of 0.5 mg/L erava susceptibility level among MRSA was 4.35% (6/138), which is markedly lower than the frequency reported with MSSA of 16.84%(32/190). Moreover, the 1.0 mg/L erava susceptibility level was not detected at all among MRSA, but detected with 2 isolates (1.58%) among MSSA (see Tables 1 and 2). Hence, MSSA were four times as likely as MRSA to exhibit an erava MIC ≥0.5 mg/L (the adopted MIC susceptibility breakpoint).

**Table 1:** Relationship of Antibiotic susceptibility with Erava MIC value levels in MRSA isolates

Antibiotic	N	Resistance rate^a^ (%)	MIC breakpoints (mg/L)	No. isolates each level	Erava MIC (mg/L) data				
					No. isolates with each MIC				MIC_50_/MIC_90_
					≤0.125	0.25	0.5	1.0	
*Total*	*138*				*54*	*78*	*6*	*0*	*0.25/0.25*
Amikacin	134	47.76	≤16	70	33	33	4	0	0.25/0.25
			32	3	0	3	0	0	0.25/0.25
			≥64	61	19	41	1	0	0.25/0.5
Erythromycin	137	95.62	≤0.5	6	4	2	0	0	0.25/0.25
			1–4	1	0	1	0	0	0.25/0.25
			≥8	130	50	75	5	0	0.25/0.25
Ciprofloxacin	133	50.38	≤1	66	32	31	3	0	0.25/0.25
			2	1	0	1	0	0	0.25/0.25
			≥4	66	19	45	2	0	0.25/0.25
Rifampicin	135	16.30	≤1	113	45	64	4	0	0.25/0.25
			≥4	22	8	14	0	0	0.25/0.25
Tetracycline	138	65.94	≤4	47	31	12	4	0	0.125/0.25
			8	17	3	14	0	0	0.25/0.25
			≥16	74	20	52	2	0	0.25/0.25
Tobramycin	133	50.38	≤4	66	32	31	3	0	0.25/0.25
			≥16	67	19	46	2	0	0.25/0.25
Nitrofurantoin	136	2.94	≤32	132	51	76	5	0	0.25/0.25
			64	2	2	0	0	0	0.125/0.25
			≥128	2	0	2	0	0	0.25/0.25
Quinupristin	124	2.42	≤1	121	47	71	3	0	0.25/0.25
			2	1	1	0	0	0	0.125
			≥4	2	1	1	0	0	0.0625/0.125

^a^The upper two MIC levels were counted as resistant for resistance rates

**Table 2:** Relationship of Antibiotic susceptibility with Erava MIC value levels in MSSA isolates.

Antibiotic	N	Resistance rate^a^ (%)	MIC breakpoints (mg/L)	No. isolates each level	Erava MIC (mg/L) data				
					No. isolates with each MIC				MIC_50_/MIC_90_
					≤0.125	0.25	0.5	1.0	
*Total*	*190*			*189*	*92*	*66*	*29*	*3*	*0.25/0.5*
Amikacin	182	3.85	≤16	174	85	59	28	3	0.25/0.5
			32	5	3	2	0	0	0.125/0.25
			≥64	2	2	0	0	0	ND
Erythromycin	186	73.66	≤0.5	48	43	1	4	0	0.125/0.5
			1–4	3	1	1	0	1	0.25/1
			≥8	134	46	63	24	2	0.25/0.5
Ciprofloxacin	181	8.84	≤1	164	93	49	20	2	0.125/0.5
			2	1	0	1	0	0	ND
			≥4	15	5	2	8	1	0.25/0.5
Rifampicin	185	4.32	≤1	176	88	61	26	2	0.25/0.5
			≥4	8	2	3	2	1	0.25/0.5
Tetracycline	188	39.89	≤4	112	76	27	8	1	0.125/0.25
			8	11	4	5	2	0	0.25/0.5
			≥16	64	10	34	19	2	0.25/0.5
Tobramycin	172	39.53	≤4	103	60	29	13	1	0.125/0.5
			8	1	0	0	1	0	ND
			≥16	67	24	28	14	2	0.25/0.5
Quinupristin	161	1.86	≤1	157	78	50	28	2	0.125/0.5
			2	1	0	1	0	0	ND
			≥4	2	1	1	0	0	ND

^a^The upper two MIC levels were counted as resistant for resistance rates

*ND*, not determined for groups with ≤2 isolates

As expected, Tet-, erythromycin-, and ciprofloxacin-resistance rates were higher among MRSA (Table 1) than among MSSA (Table 2). Looking at the intersectionality of susceptibilities to these antibiotics with intermediate-resistance status and erava MIC values, it is noteworthy that erava MICs ≥0.5 mg/L were rare among MRSA isolates but relatively more frequent among MSSA isolates. That is, among 74 Tet-resistant MRSA, only 2.7% (2/74) had an erava MIC ≥0.5 mg/L, whereas among 64 Tet-resistant MSSA, 32.8% (21/64) had an erava MIC ≥0.5 mg/L (Data in Tables 1 and 2). Similarly, 3.9%(5/130) and 19.4% (26/134) of the erythromycin-resistant MRSA and MSSA, respectively, had an erava MIC ≥0.5 mg/L; and 60%(9/15) and 3.0%(2/66) of the ciprofloxacin -resistant MSSA and MRSA, respectively, had an erava MIC ≥0.5 mg/L (Data in Tables 1 and 2).

### Clonality of erava MIC distribution in clinical *S. aureus* isolates

The ST distributions determined by MLST among our MRSA and MSSA isolates were shown in Additional file 1: Tables S1 and S2, respectively. We identified 18 STs among the MRSA isolates, with the predominant STs being ST239, ST59, and ST1 (Additional file 1: Table S2). We identified 34 STs among the MSSA, with the predominant STs being ST7, ST59, ST398, and ST188 (Additional file 1: Table S3). MRSA showed robust clustering within their predominant STs, with 79.7% (110/ 138) of MRSA belonging to the predominant STs. MSSA showed weaker clustering within their predominant STs, with only 45.3%(86/ 190) MSSA belonging of the predominant STs.

The distributions of Tet resistance and erava-breakpoint MIC levels among the aforementioned predominant MRSA and MSSA STs are reported in Table 3. Note that 83.3%(5/6) of the total MRSA isolates that had an erava MIC ≥0.5 mg/L (83.3%) belonged to the top two MRSA STs. Conversely, only 35.5%(12/31) of the total MSSA isolates that had an erava MIC ≥0.5 mg/L (41.94%) gathered to the aforementioned top five STs.

**Table 3:** Erava MIC values of predominant *S. aureus* STs

MLST	N	Tetracycline, n						Erava, n			
		Isolates with each MIC (mg/L)				MIC_50_/MIC_90_	Isolates with each MIC (mg/L)				MIC_50_/MIC_90_
		≤0.125	0.25	0.5	1.0		≤0.125	0.25	0.5	1.0	
*MRSA*											
ST239	62	6	0	3	53	> 8/> 8	15	45	2	0	0.25/0.25
ST59	41	18	1	12	10	8/> 8	19	19	3	0	0.25/0.25
ST1	7	6	0	0	1	≤0.5/≤0.5	6	1	0	0	0.0625/0.125
*MSSA*											
ST7	35	12	1	1	21	> 8/> 8	12	21	2	0	0.25/0.25
ST59	22	10	1	6	5	4/> 8	12	8	1	1	0.125/0.25
ST398	16	8	0	0	8	≤0.5/> 8	8	1	7	0	0.125/0.5
ST239	13	12	0	0	1	≤0.5/≤0.5	9	4	0	0	0.125/0.25
ST88	7	3	0	0	4	≤0.5/> 8	4	2	1	0	0.125/0.25

There was a notable divergence in erava susceptibility between MRSA and MSSA within STs. For example, 7.32% (3/41) of ST59-MRSA and 9.1% (2/22) of ST59-MSSA had an erava MIC ≥0.5 mg/L. However, 43.8% of ST398-MSSA (7/16) had an erava MIC ≥0.5 mg/L, suggesting that ST398 may have a tendency favoring the development of erava resistance.

### Antimicrobial susceptibility of erava in clinical *S. aureus* isolates harboring Tet specific resistance factors

The frequencies of clinical *S. aureus* isolates harboring the genes that encode Tet(M), Tet(K), and Tet(L), overall and in combination, are reported in Table 4. Tet(O) is omitted from Table 4 because we did not find any isolates with Tet(O)*.* Note that total 69 MRSA isolates and 73 MSSA isolates were detected positively with Tet specific resistance genes in this study. Tet(K) alone was the most common Tet specific resistance gene in MSSA and its frequency was 33.2%(63/190), with far lower proportions of isolates harboring tet (L) alone (1.1%) and tet (M) alone (1.1%) respectively (data in Table 4). Whereas in MRSA, the frequency of isolates with tet (M) and Tet (K) alone was both 19.6%(27/138) respectively and tet (L) alone is also seldomly detected in MRSA (data in Table 4).

**Table 4:** In vitro activity of erava against S aureus with Tet specific resistant genes

Tet resistance factors	N	Tetracycline			Erava					
		No. isolates with each MIC (mg/L)			No. isolates with each MIC (mg/L)				MIC range	MIC_50_/MIC_90_
		≤4.0	8.0	≥16.0	≤0.125	0.25	0.5	1.0		
*MRSA*										
Tet(M)	27	1	1	25	10	17	0	1	≤0.125–0.25	0.25/0.25
Tet(K)	27	3	8	16	2	24	3	0	0.0625–0.5	0.25/0.5
Tet(L)	5	0	1	4	1	4	0	0	0.125–0.25	0.25/0.25
Tet(M), Tet(L)	4	0	1	3	0	4	0	0	0.25	0.25/0.25
Tet(L), Tet(K)	3	0	3	0	2	1	0	0	0.125–0.25	ND
Tet(M), Tet(K)	2	0	0	2	0	1	1	0	0. 25–0.5	ND
Tet(M), Tet(L), Tet(K)	1	0	0	1	1	0	0	0	0.0625	ND
-^a^	69	43	3	23	38	29	2	0	0.0625–0.5	0.125/0.25
*MSSA*										
Tet(M)	2	2	0	0	1	1	0	0	≤0.125–0.25	ND
Tet(K)	63	14	10	39	12	33	17	1	0.0625–1.0	0.25/0.5
Tet(L)	2	0	0	2	0	0	2	0	0.5	ND
Tet(L), Tet(K)	5	1	0	4	1	0	3	1	0.125–1.0	0.5/0.5
Tet(M), Tet(K)	1	1	0	0	0	0	1	0	0.5	ND
-^a^	115	94	1	20	76	32	6	1	0.0625–1.0	0.125/0.5

-^a^ negative for tetracycline resistance genes; ND, not determined for groups with ≤3 isolates

The MIC_90_ values for MSSA isolates harboring Tet(M), Tet(L), or Tet(K) were 0.5 mg/L, whereas those for the MRSA isolates with Tet specific resistance genes were predominantly 0.25 mg/L, demonstrating generally strong in vitro activity of erava against Tet specific resistance factor-carrying *S. aureus*, especially MRSA. Whereas, 12.1% (4/33)of the MRSA isolates with Tet(K) alone or combination and 33.3% (23/69)of the MSSA isolates with Tet(K) alone or combination were shown with erava MICs≥0.5 mg/L (data in Table 3). Concerningly, of the 32 MSSA with MICs≥0.5 mg/L, 23 isolates carried tet (K) alone or combination. Meanwhile, a divergence between MRSA and MSSA was observed with respect to the relationship between tet (L)-positive isolates, wherein 2/2 (100%) of MSSA carrying tet (L) alone and 80%(4/5) of tet (L) combination had MICs ≥0.5 mg/L and None of total 13 isolates with tet (L) alone or combination was shown with MICs ≥0.5 mg/L, demonstrating comparatively high MICs among tet (L)-positive MSSA isolates. Conversely, erava MICs≥0.5 mg/L among tet (M)-carrying isolates were relatively rare compared with tet (L), especially worthy of concern, with only 2/34 (5.9%) of MRSA carrying tet (M) alone or conbination and 1/3 (33.3%) of MSSA carrying tet (M) alone or combination exhibiting an erava MIC≥0.5 mg/L (Table 3).

### Erava heteroresistance in *S. aureus*

The erava heteroresistance test results and heteroresistant clone characteristics are reported in Table 5. Two clones picked from each plate of the heteroresistant subpopulation were assessed and the erava MIC values of the heteroresistance-derived clones were in the range of 1–4 mg/L. Our heteroresistance analysis indicated that the MRSA and MSSA mother isolates with erava MICs of ≤0.125 mg/L and 0.25 mg/L did not exhibit erava heteroresistance. Erava heteroresistance were found in 13.79% (4/29) of MSSA mother isolates, but no MRSA mother isolates (0.0% of 6), with an erava MIC = 0.5 mg/L. The tigecycline MICs in the erava heteroresistance-derived MSSA clones suggested that they tended to have greater tigecycline MIC creep than erava MIC creep (Table 4).

**Table 5:** Characteristics of clinical heteroresistant mother *S. aureus* strains and characteristics of heteroresistance-derived *S. aureus* clones

Mother strains		Characteristics of heteroresistance-derived clones							
Class / Tet factor	Erava MIC	Clone strain ID	Erava MICs (mg/L)			Tigecycline MICs (mg/L)			30S ribosomal subunit mutations^a^
			Alone	+CCP	+PAβN	Alone	+CCP	+PAβN	
MSSA / tet(L)	0.5	CHS237-E1	2	≤0.03	0.25	16	≤0.03	0.5	None
		CHS237-E2	2	≤0.03	0.25	2	≤0.03	0.5	None
MSSA / none	0.5	CHS632-E1	1	≤0.03	0.25	4	≤0.03	0.25	None
		CHS632-E2	2	≤0.03	0.25	4	≤0.03	0.25	None
MSSA / none	0.5	CHS62-E1	1	≤0.03	0.25	8	≤0.03	0.5	None
		CHS62-E2	1	≤0.03	0.25	4	≤0.03	0.25	None
MSSA/tet(K) + tet(L)	0.5	CHS239-E1	4	≤0.03	0.25	4	≤0.03	0.5	None
		CHS239-E2	1	≤0.03	0.25	4	≤0.03	0.5	None

^a^Including mutations in five 16S ribosomal gene copies and genes encoding the 30S ribosomal proteins S3 and S10

No genetic mutations affecting the 30S ribosomal subunits (five 16SrRNA gene copies and the 30S ribosomal proteins S3 and S10) were found in the heteroresistance -derived clones (Table 5), nor were any found among the three clinical MSSA mother isolates with erava MICs of 1.0 mg/L (Additional file 1: Table S4). Relative to the data obtained in the absence of an efflux pump inhibitor, the erava MICs of all *S. aureus* heteroresistance-derived isolates (range, 1.0–4.0 mg/L) were markedly reduced in the presence of CCCP (to ≤0.03 mg/L) and in the presence of PaβN (to 0.25 mg/L)(Table 5). The efflux pump inhibitors had similar effects on the tigecycline MIC values of these heteroresistance-derived clones. The erava MIC values of clinical MSSA isolates with erava MICs of 1 mg/L were also decreased to ≤0.03 mg/L by CCCP and to 0.25 mg/L by PaβN (Additional file 1: Table S4).

## Discussion

We observed excellent in vitro erava efficacy against all 328 clinical *S. aureus* isolates from China examined in this study, with erava MIC_50_/MIC_90_ values of 0.25/0.25 mg/L for MRSA and 0.25/0.5 mg/L for MSSA. These findings are consistent with previous reports indicating that *staphylococci*, *streptococci*, and *enterococci*, regardless of concurrent resistance phenotypes, have typically been found to have erava MIC_50_ and MIC_90_ values ≤0.25 mg/L, with no more than a two-fold MIC_50_ to MIC_90_ difference [3–5]. The highest erava MICs reported previously for clinical MRSA and MSSA isolates were 4.0 mg/L and 0.5 mg/L, respectively, with higher-end values being found for hospital-acquired MRSA, as opposed to community-acquired MRSA and MSSA [3]. Relative to our MRSA isolates, our MSSA isolates had a lower frequency of Tet resistance, but a higher frequency of isolates with erava MICs ≥0.5 mg/L. Moreover, our findings of both MRSA and MSSA isolates with erava MICs ≥0.5 mg/L, and of a few MSSA isolates with erava MICs of 1 mg/L indicate higher erava MICs among MSSA than MRSA from this region and are especially worthy of concern.

The aforementioned erava MIC data differ from the reported data previously showing higher erava MICs in MRSA than MSSA [3, 4]. These differences could be due, at least in part, to sample and regional variation given that the molecular and antimicrobial susceptibility characteristics of *S. aureus* are known to vary across regionsand the very limited volume of data that have been reported regarding erava effects on *S. aureus* [28, 29]. Previously, there has been a focus on the antimicrobial susceptibility of erava mainly in the multi-drug resistant *S. aureus*, including MRSA as well as vancomycin-resistant and linezolid-resistant isolates. Our sample did not include vancomycin-resistant or linezolid-resistant *S. aureus* due to their low frequency in China; however, we did encounter a high frequency of erythromycin- resistant *S. aureus*. Further comparative studies of the antimicrobial activity of erava in MRSA versus MSSA are needed.

Recent reports have suggested that vancomycin, teicoplanin, daptomycin, and linezolid MIC creep in MRSA may be associated with clonality [29–31]. In the present data, erava MICs ≥0.5 mg/L were strongly represented among ST239 and ST59 MRSA isolates, but generally diversified among MSSA isolates though relatively well represented among ST398-MSSA isolates. The relationship of *S. aureus* clonality with erava susceptibility should be examined in large samples across different regions.

Recent evidence indicates that new-generation drugs of Tet class, including tigeycline and erava, can overcome the Tet-specific resistance mechanisms, including the efflux pumps such as Tet(K) and Tet(L) and the ribosome protection protein such as Tet(M). Tet(K) and Tet(L), constituted by 14 transmembrane segments as monovalent cation-H^+^ antiporters, are the most common Tet-specific efflux pumps in clinical Gram-positive isolates and play an important role in microbial coping with alkali stress, sodium stress, and potassium insufficiency [8]. Tet(M), a common and well-characterized ribosomal protection protein, catalyzes the GTP-dependent release of Tet from ribosomes [8]. Worthy of our concern, a recent study has linked tigecycline resistance in *E. faecalis* to Tn916-associated constitutive overexpression and increased copy numbers of Tet(M) and Tet(L) [14]. In this context, it is noteworthy that our data showed good in vitro activity of erava against clinical *S. aureus* isolates harboring Tet(M), Tet(L) and Tet(K) in both MRSA and MSSA, indicating that erava has the potential to overcome the common tetracycline specific resistance mechanisms. Moreover, our data showed a disassociation between Tet resistance trends and erava MIC creep.

Erava, being a new Tet class drug, has not yet completed its phase III clinical trial and does not yet have its own susceptibility breakpoints recommended by CLSI [3–7, 24]. Consequently, we referred conservatively to the tigecycline susceptibility MIC breakpoint for *S. aureus* of 0.5 mg/L, which was derived from the US Food and Drug Administration [24–26, 28]. The available data thus far suggest that erava is two- to four-fold more active than tigecycline against common clinically important Gram-positive aerobic species [3, 4]. Because erava is not yet in use clinically in China and erava MICs of 0.5 mg/L were observed often in both MRSA and MSSA, we hypothesized that all of these *S. aureus* isolates would be susceptible to erava, and thus we referred to the MIC susceptibility breakpoint to define heteroresistant mother strains with subpopulations able to grow in the presence of 1.0 mg/L erava. Evaluation of heteroresistance is useful for probing antimicrobial resistance risk. None of the MSSA isolates with erava MIC values of ≤0.125 or 0.25 met our criteria for heteroresistance, whereas we did observe heteroresistance risk among MSSA with erava MIC values of 0.5 mg/L, suggesting that we should be on alert for erava MIC creep and the potential emergence of erava resistance. Although no MRSA were found to be exhibiting erava heteroresistance, it should be noted that six MRSA isolates had erava MIC values of 0.5 mg/L, underscoring the need for further screening of wider samples of MRSA isolates for erava heteroresistance.

Like other Tet class, erava inhibits bacterial protein synthesis by binding to the 30S ribosomal subunit [3, 8], and genetic mutations affecting the 30S ribosomal subunit (i.e., 16SrRNA and ribosome proteins S3 and S10) have been shown to confer resistance to tigecycline [3, 12–20]. Although we found heteroresistance-derived clones with elevated erava MICs and clinical MSSA with MIC values of 1.0 mg/L, none of these clones had 30S ribosomal subunit mutations. The potential relationship between Tet target site mutations with erava heteroresistance and relatively high MICs in *S. aureus* warrants further exploration given the ample evidence linking tigecycline heteroresistance in Gram-negative bacteria to cell envelope and efflux pump proteins [10, 21, 23]. The presently observed suppressing effects of PAβN and CCCP on the erava and tigecycline MICs of heteroresistance-derived *S. aureus* clones and clinical isolates with MICs of 1.0 mg/L suggest that efflux pumps and cell envelopes also contribute to erava heteroresistance in *S. aureus* [21–23]. Importantly, our finding of higher MICs for tigecycline than for erava in erava heteroresistance-derived clones is suggestive of possible tigecycline cross-resistance. Furthermore, the suppressing effects of CCCP and PAβN on tigecycline MICs further implicates efflux pump/cell envelope components in tigecycline MIC creep.

Upregulation of MepA, a multidrug-resistant efflux pump, has been shown to confer tigecycline resistance in *S. aureus* [16–18]. Furthermore, in strain SA984 *S. aureus*, the erava MIC increased from 0.004 mg/L in a MepA-negative parent isolate to 0.016 mg/L in *S. aureus* expressing MepA, whereas MepA addition increased tigecycline MICs from 0.016 mg/L to 1.0 mg/L, pointing to a negligible effect of MepA on erava resistance relative to its effect on tigecycline resistance [3, 8, 16]. The molecular mechanisms by which efflux pumps and cell envelope components participate in the MIC creep of these new Tet class drugs need to be further studied.

## Conclusions

In conclusion, erava exhibited excellent in vitro activity against clinical *S. aureus* isolates from China, overcoming the presence of Tet factors, including the Tet(K) and Tet(L) efflux pumps and the ribosome protection protein Tet(M). However, the risk of erava heteroresistance in *S. aureus*, especially in MSSA with MICs ≥0.5 mg/L, is worthy of concern. MSSA had higher MICs and a higher frequency of erava heteroresistance than MRSA in this study. *S. aureus* with erava MICs of 1.0 mg/L did not have 30S ribosome subunit mutations and the MICs of heteroresistance-derived MSSA and MSSA with erava MICs ≥1.0 mg/L could be reduced by CCCP or PAβN. Further studies are needed to elucidate the mechanisms by which efflux pump and cell envelope proteins may enable erava heteroresistance development and MIC creep in *S. aureus*.

## Additional files

Additional file 1: **Table S1.** Primers used to detect Tet-resistance genes and 30S ribosome subunits in *S. aureus* by PCR. **Table S2.** MLST-determined ST distribution among MRSA. **Table S3.** MLST-determined ST distribution among MSSA. **Table S4.** Characteristics of MSSA isolates with erava MICs of 1.0 mg/L. (DOCX 20 kb)

## Abbreviations

CCCP: Carbonyl cyanide m-chlorophenylhydrazine
Erava: Erevacycline
MICs: Minimum inhibitory concentrations
MLST: Multilocus seque nce typing
MRSA: Methicillin-resistant *Sthaphylococcus aureas*
MSSA: Methecillin-sensitive *Sthaphylococcus aureas*
PAP: Population analysis profiling
PAβN: Phe-Arg-β- naphthylamide
PCR: Polymerase chain reaction
*S. aureus*: *Staphylococcus aureus*
STs: Sequence types
Tet: Tetracycline

## References

[1] Smith JR, Frens JJ, Snider CB, Claeys KC (2018). Impact of a pharmacist-driven care package on Staphylococcus aureus bacteremia management in a large community healthcare network: a propensity score-matched, quasi-experimental study. Diagn Microbiol Infect Dis.

[2] Bal AM, David MZ, Garau J, Gottlieb T, Mazzei T, Scaglione F, Tattevin P, Gould IM (2017). Future trends in the treatment of methicillin-resistant Staphylococcus aureus (MRSA) infection: an in-depth review of newer antibiotics active against an enduring pathogen. J Glob Antimicrob Resist.

[3] Zhanel GG, Cheung D, Adam H, Zelenitsky S, Golden A, Schweizer F, Gorityala B, Lagace-Wiens PR, Walkty A, Gin AS, Hoban DJ, Karlowsky JA (2016). Review of Eravacycline, a novel Fluorocycline antibacterial agent. Drugs.

[4] Thabit AK, Monogue ML, Newman JV, Nicolau DP (2018). Assessment of in vivo efficacy of eravacycline against Enterobacteriaceae exhibiting various resistance mechanisms: a dose-ranging study and pharmacokinetic/ pharmacodynamic analysis. Int J Antimicrob Agents.

[5] Monogue ML, Thabit AK, Hamada Y, Nicolau DP (2016). Antibacterial efficacy of Eravacycline in vivo against gram-positive and gram-negative organisms. Antimicrob Agents Chemother.

[6] Snydman DR, McDermott LA, Jacobus NV, Kerstein K, Grossman TH, Sutcliffe JA (2018). Evaluation of the in vitro activity of Eravacycline against a broad Spectrum of recent clinical anaerobic isolates. Antimicrob Agents Chemother.

[7] Sutcliffe JA, O'Brien W, Fyfe C, Grossman TH (2013). Antibacterial activity of eravacycline (TP-434), a novel fluorocycline, against hospital and community pathogens. Antimicrob Agents Chemother.

[8] Nguyen F, Starosta AL, Arenz S, Sohmen D, Donhofer A, Wilson DN (2014). Tetracycline antibiotics and resistance mechanisms. Biol Chem.

[9] Claeys KC, Lagnf AM, Hallesy JA, Compton MT, Gravelin AL, Davis SL, Rybak MJ (2016). Pneumonia caused by methicillin-resistant Staphylococcus aureus: does vancomycin Heteroresistance matter?. Antimicrob Agents Chemother.

[10] Chen Y, Hu D, Zhang Q, Liao XP, Liu YH, Sun J (2017). Efflux pump overexpression contributes to Tigecycline Heteroresistance in Salmonella enterica serovar typhimurium. Front Cell Infect Microbiol.

[11] Zhong X, Xu H, Chen D, Zhou H, Hu X, Cheng G (2014). First emergence of acrAB and oqxAB mediated tigecycline resistance in clinical isolates of Klebsiella pneumoniae pre-dating the use of tigecycline in a Chinese hospital. PLoS One.

[12] Linkevicius M, Sandegren L, Andersson DI (2016). Potential of tetracycline resistance proteins to evolve Tigecycline resistance. Antimicrob Agents Chemother.

[13] Grossman TH (2016). Tetracycline antibiotics and resistance. Cold Spring Harb Perspect Med.

[14] Fiedler S, Bender JK, Klare I, Halbedel S, Grohmann E, Szewzyk U, Werner G (2016). Tigecycline resistance in clinical isolates of enterococcus faecium is mediated by an upregulation of plasmid-encoded tetracycline determinants tet(L) and tet(M). J Antimicrob Chemother.

[15] Argudín MA, Roisin S, Dodémont M, Nonhoff C, Deplano A, Denis O (2017). Mutations at the ribosomal S10 gene in clinical strains of Staphylococcus aureus with reduced susceptibility to Tigecycline. Antimicrob Agents Chemother.

[16] Dabul ANG, Avaca-Crusca JS, Van Tyne D, Gilmore MS, Camargo I (2018). Resistance in in vitro selected Tigecycline-resistant methicillin-resistant Staphylococcus aureus sequence type 5 is driven by mutations in mepR and mepA genes. Microb Drug Resist.

[17] Seriki AT, Smith SI, Adeleye AI, Fowora MA (2018). Molecular analysis of low-level tetracycline resistance in clinical isolates of helicobacter pylori among dyspeptic patients in south West Nigeria. J Glob Antimicrob Resist.

[18] Haim MS, Di Gregorio S, Galanternik L, Lubovich S, Vazquez M, Bharat A, Zaheer R, Golding GR, Graham M, Van Domselaar G, Cardona ST, Mollerach M (2017). First description of rpsJ and mepA mutations associated with tigecycline resistance in Staphylococcus aureus isolated from a cystic fibrosis patient during antibiotic therapy. Int J Antimicrob Agents.

[19] Niebel M, Quick J, Prieto AM, Hill RL, Pike R, Huber D, David M, Hornsey M, Wareham D, Oppenheim B, Woodford N, van Schaik W, Loman N (2015). Deletions in a ribosomal protein-coding gene are associated with tigecycline resistance in enterococcus faecium. Int J Antimicrob Agents.

[20] Cattoir V, Isnard C, Cosquer T, Odhiambo A, Bucquet F, Guerin F, Giard JC (2015). Genomic analysis of reduced susceptibility to tigecycline in enterococcus faecium. Antimicrob Agents Chemother.

[21] Rampioni G, Pillai CR, Longo F, Bondi R, Baldelli V, Messina M, Imperi F, Visca P, Leoni L (2017). Effect of efflux pump inhibition on Pseudomonas aeruginosa transcriptome and virulence. Sci Rep.

[22] Osei Sekyere J, Amoako DG (2017). Carbonyl cyanide m-Chlorophenylhydrazine (CCCP) reverses resistance to Colistin, but not to Carbapenems and Tigecycline in multidrug-resistant Enterobacteriaceae. Front Microbiol.

[23] Halaby T, Kucukkose E, Janssen AB, Rogers MR, Doorduijn DJ, van der Zanden AG, Al Naiemi N, Vandenbroucke-Grauls CM, van Schaik W (2016). Genomic characterization of Colistin Heteroresistance in Klebsiella pneumoniae during a nosocomial outbreak. Antimicrob Agents Chemother.

[24] Clinical and Laboratory Standards Institute (2016). Performance standards for antimicrobial susceptibility testing: 24th informational supplement. Document M100-S26.

[25] Pfizer. Tygacil Package Insert. Available from: http://labeling.pfizer.com/ShowLabeling.aspx?id=491#section-6.1; 2016. [Accessed 23 February 2017].

[26] Pfaller MA, Huband MD, Streit JM, Flamm RK, Sader HS (2018). Surveillance of tigecycline activity tested against clinical isolates from a global (North America, Europe, Latin America and Asia-Pacific) collection (2016). Int J Antimicrob Agents.

[27] Bai B, Hu K, Li H, Yao W, Li D, Chen Z, Cheng H, Zheng J, Pan W, Deng M, Liu X, Lin Z, Deng Q, Yu Z. Effect of tedizolid on clinical enterococcus isolates: in vitro activity, distribution of virulence factor, resistance genes and multilocus sequence typing. FEMS Microbiol Lett. 2018;365(3). 10.1093/femsle/fnx284. 10.1093/femsle/fnx284 29390078

[28] Tang YT, Cao R, Xiao N, Li ZS, Wang R, Zou JM, Pei J (2018). Molecular epidemiology and antimicrobial susceptibility of methicillin-resistant Staphylococcus aureus isolates in Xiangyang. China J Glob Antimicrob Resist.

[29] Tegegne HA, Kolackova I, Karpiskova R (2017). Diversity of livestock associated methicillin-resistant Staphylococcus aureus. Asian Pac J Trop Med.

[30] Doudoulakakis A, Spiliopoulou I, Spyridis N, Giormezis N, Kopsidas J, Militsopoulou M, Lebessi E, Tsolia M (2017). Emergence of a Staphylococcus aureus clone resistant to mupirocin and Fusidic acid carrying exotoxin genes and causing mainly skin infections. J Clin Microbiol.

[31] Hsieh YC, Lin YC, Huang YC (2016). Vancomycin, teicoplanin, daptomycin, and linezolid MIC creep in methicillin-resistant Staphylococcus aureus is associated with clonality. Medicine (Baltimore).

