# Supplementary material for: Eravacycline activity against clinical S. aureus isolates from China: in vitro activity, MLST profiles and heteroresistance
Source: BMC Microbiol. 2018 Dec 13;18:211. doi: 10.1186/s12866-018-1349-7 (PMC6293590; doi:10.1186/s12866-018-1349-7)
Supplement: Supplementary file 1 — Table S1. Primers used to detect Tet-resistance genes and 30S ribosome subunits in S. aureus by PCR. Table S2. MLST-determined ST distribution among MRSA. Table S3. MLST-determined ST distribution among MSSA. Table S4. Characteristics of MSSA isolates with erava MICs of 1.0 mg/L. (DOCX 20 kb) [file 12866_2018_1349_MOESM1_ESM.docx]

**Table S1 Primers used to detect Tet-resistance genes and 30S ribosome subunits**

**in *S. Aureus* by PCR.**

| **Target gene** | **Primer** | **Primer sequence (5'-3')** | **Amplicon size (bp)** | **Reference** |
| --- | --- | --- | --- | --- |
| *tet*(M) | tet(M)-F | CAATACAATAGGAGCAAGC | 974 | bai etal 2018 |
|  | tet(M)-R | CGAACAAGAGGAAAGCATAAG |  |  |
| *tet*(L) | tet(L)-F | GTAACCAGCCAACTAATGAC | 908 | bai etal 2018 |
|  | tet(L)-R | TTGGATCGATAGTAGCC |  |  |
| *tet*(K) | tet(K)-F | TCGATAGGAACAGCAGTA | 139 | bai etal 2018 |
|  | tet(K)-R | CAGCAGATCCTACTCCTT |  |  |
| *tet*(O) | tet(O)-F | AACTTAGGCATTCTGGCTCAC | 515 | bai etal 2018 |
|  | tet(O)-R | TCCCACTGTTCCATATCGTCA |  |  |
| 16SrRNA-RR1 | RR1-F | ATATGTCACGTTATTCCGCATCTTC | 2086 | In this study |
|  | RR1-R | GCGGTGTTTTGAGAGATTATTTA |  |  |
| 16SrRNA-RR2 | RR2-F | ATATGTCACGTTATTCCGCATCTTC | 2075 | In this study |
|  | RR2-R | GCAGACGCACAGGACTTC |  |  |
| 16SrRNA-RR3 | RR3-F | ATATGTCACGTTATTCCGCATCTTC | 1936 | In this study |
|  | RR3-R | GTCGTCAAACGGCACTAATA |  |  |
| 16SrRNA-RR4 | RR4-F | ATATGTCACGTTATTCCGCATCTTC | 1756 | In this study |
|  | RR4-R | ATCACCCGCTCCATAGATAAT |  |  |
| 16SrRNA-RR5 | RR5-F | ATATGTCACGTTATTCCGCATCTTC | 2345 | In this study |
|  | RR5-R | AGGTGCGATGGCAAAACA |  |  |
| SA30S-S3 | S3-R | GCAGATTCGATTTGACGAGAT | 810 | In this study |
|  | S3-F | ACGGTAAAGAAGAAGCTAAAG |  |  |
| SA-S10 | S10-R | CTCGAAAATAGTTGAACTGACTAAG | 1920 | In this study |
|  | S10-F | TTCAGAAGATTTCTCAGTGATTACG |  |  |

References: [1]. Bai B, Hu K, Li H, Yao W, Li D, Chen Z, Cheng H, Zheng J, Pan W, Deng M, Liu X, Lin Z, Deng Q, Yu Z. Effect of tedizolid on clinical *Enterococcus* isolates: in vitro activity, distribution of virulence factor, resistance genes and multilocus sequence typing. FEMS Microbiol Lett. 2018 Feb 1;365(3). doi: 10.1093/femsle/ fnx284.

**Table S2. MLST-determined ST distribution among MRSA**.

| **ST** | **No. isolates** | **Portion, %** |
| --- | --- | --- |
| ST239 | 62 | 44.93 |
| ST59 | 41 | 29.71 |
| ST1 | 7 | 5.07 |
| ST188 | 4 | 2.90 |
| ST3191 | 2 | 1.45 |
| ST338 | 2 | 1.45 |
| ST5 | 2 | 1.45 |
| ST1821 | 1 | 0.72 |
| ST237 | 1 | 0.72 |
| ST238 | 1 | 0.72 |
| ST2631 | 1 | 0.72 |
| ST455 | 1 | 0.72 |
| ST10 | 1 | 0.72 |
| ST630 | 1 | 0.72 |
| ST7 | 1 | 0.72 |
| ST88 | 1 | 0.72 |
| ST942 | 1 | 0.72 |
| Not typed | 9 | 5.80 |
| *Total* | *138* | *100* |

**Table S3. MLST-determined ST distribution among MSSA**.

| **ST** | **No. isolates** | **Portion, %** |
| --- | --- | --- |
| ST7 | 35 | 18.42 |
| NT | 27 | 14.21 |
| ST59 | 22 | 11.58 |
| ST398 | 16 | 8.42 |
| ST188 | 13 | 6.84 |
| ST120 | 7 | 3.68 |
| ST6 | 7 | 3.68 |
| ST88 | 7 | 3.68 |
| ST25 | 5 | 2.63 |
| ST30 | 5 | 2.63 |
| ST5 | 5 | 2.63 |
| ST1 | 4 | 2.11 |
| ST633 | 4 | 2.11 |
| ST15 | 3 | 1.58 |
| ST239 | 3 | 1.58 |
| ST72 | 3 | 1.58 |
| ST965 | 3 | 1.58 |
| ST2114 | 2 | 1.05 |
| ST217 | 2 | 1.05 |
| ST2483 | 2 | 1.05 |
| ST630 | 2 | 1.05 |
| ST1036 | 1 | 0.53 |
| ST121 | 1 | 0.53 |
| ST1281 | 1 | 0.53 |
| ST1821 | 1 | 0.53 |
| ST1921 | 1 | 0.53 |
| ST20 | 1 | 0.53 |
| ST2797 | 1 | 0.53 |
| ST2871 | 1 | 0.53 |
| ST363 | 1 | 0.53 |
| ST8 | 1 | 0.53 |
| ST861 | 1 | 0.53 |
| ST943 | 1 | 0.53 |
| ST950 | 1 | 0.53 |
| *Total* | *190* | *100* |

**Table S4. Characteristics of MSSA isolates with erava MICs of 1.0 mg/L.**

| **No.** | **ST** | **Tet resistance factor** | **MICs (mg/L)** | | | **30S ribosomal subunit mutations*** |
| --- | --- | --- | --- | --- | --- | --- |
|  |  |  | **Tet** | **Erava**  **+CCP** | **Erava**  **+PAβN** |  |
| 1 | ST2797 | Tet(K)+tet(L) | ≤0.5 | ≤0.03 | 0.25 | None |
| 2 | ST59 | None | >8.0 | ≤0.03 | 0.25 | None |
| 3 | ST633 | Tet(K) | >8.0 | ≤0.03 | 0.25 | None |

Only three *S. aureus* strains had an erava MIC = 1.0, including three MSSA and zero MRSA.

*Including mutations in five 16S ribosomal gene copies and genes encoding the 30S ribosomal proteins S3 and S10.
